# Association Mapping for Epistasis and Environmental Interaction of Yield Traits in 323 Cotton Cultivars under 9 Different Environments

**DOI:** 10.1371/journal.pone.0095882

**Published:** 2014-05-08

**Authors:** Yinhua Jia, Xiwei Sun, Junling Sun, Zhaoe Pan, Xiwen Wang, Shoupu He, Songhua Xiao, Weijun Shi, Zhongli Zhou, Baoyin Pang, Liru Wang, Jianguang Liu, Jun Ma, Xiongming Du, Jun Zhu

**Affiliations:** 1 Institute of Cotton Research of Chinese Academy of Agricultural Sciences (ICR, CAAS), State Key Laboratory of Cotton Biology, Key Laboratory of Cotton Genetic Improvement, Ministry of Agriculture, Anyang, China; 2 Key Laboratory of Crop Germplasm Resource of Zhejiang Province, Zhejiang University, Hangzhou, China; 3 Institute of industrial Crops, Jiangsu Academy of Agricultural Sciences, Nanjing, China; 4 Economic Crop Research Institute, Xinjiang Academy of Agricultural Science, Urumqi1, China; East Carolina University, United States of America

## Abstract

Improving yield is a major objective for cotton breeding schemes, and lint yield and its three component traits (boll number, boll weight and lint percentage) are complex traits controlled by multiple genes and various environments. Association mapping was performed to detect markers associated with these four traits using 651 simple sequence repeats (SSRs). A mixed linear model including epistasis and environmental interaction was used to screen the loci associated with these four yield traits by 323 accessions of *Gossypium hirsutum* L. evaluated in nine different environments. 251 significant loci were detected to be associated with lint yield and its three components, including 69 loci with individual effects and all involved in epistasis interactions. These significant loci explain ∼ 62.05% of the phenotypic variance (ranging from 49.06% ∼ 72.29% for these four traits). It was indicated by high contribution of environmental interaction to the phenotypic variance for lint yield and boll numbers, that genetic effects of SSR loci were susceptible to environment factors. Shared loci were also observed among these four traits, which may be used for simultaneous improvement in cotton breeding for yield traits. Furthermore, consistent and elite loci were screened with −Log_10_ (*P*-value) >8.0 based on predicted effects of loci detected in different environments. There was one locus and 6 pairs of epistasis for lint yield, 4 loci and 10 epistasis for boll number, 15 loci and 2 epistasis for boll weight, and 2 loci and 5 epistasis for lint percentage, respectively. These results provided insights into the genetic basis of lint yield and its components and may be useful for marker-assisted breeding to improve cotton production.

## Introduction

Cotton is one of the most important economic crops in the world and provides large amounts of raw materials for textile industry. High yield is always remained the primary focus of cotton breeding programs. Boll number, boll weight, and lint percentage are three yield component traits, which are complex traits controlled by multiple genes with gene × gene interaction and gene × environment interaction [1]–[2]. Understanding genetic architecture of lint yield and its component traits at the molecular level is very important for efficiently improving cotton yield traits. Identifying and charactering the key QTLs and genes affecting yield traits in cotton has been a research frontier over the past few decades. On the basis of linkage analysis in the QTL mapping populations, several QTLs controlling lint yield and its component traits have been obtained [1]–[8]. However, these detected QTLs involved in large regions of the chromosome, where candidate genes or genetic variants are difficult to distinguish. In addition, the mapping results have only limit utilization because of lower genetic diversity in the special population used for QTL mapping and can not fully elucidate the genetic basis of lint yield and its component traits.

Genome-wide association studies (GWAS), which are based on the linkage disequilibrium (LD), provide the opportunity to systematically identify the genetic components of complex traits of crops in recent years [9]–[14]. It has the potential to detect single nucleotide polymorphisms (SNPs) or other types of molecular markers such as single sequence repeats (SSRs) within or nearby a gene. It can extensively exploit historical recombination and natural genetic diversity in natural population, and overcome the limitations of conventional linkage mapping for crop breeding. Recently, Zhang *et al* performed association mapping using 81 accessions of Upland cotton based on 121 SSRs to map 12 agronomical and fiber quality traits, and identified 180 loci only with additive effects significantly associated with the traits [14]. However, current GWAS predominately focus on examination of SNPs at a single environment and fails to detect the gene × gene interaction as well as gene × environment interaction across multiple environments. The genetic variation of complex traits is contributed in part by epistasis and environmental interaction effects. Therefore, searching for only major effects may miss other key genetic variants specific to environment factors and cannot provide reliable estimates for genetic effects [3].

Due to a heavy computational burden, current methods of GWAS have not been used for detecting gene × gene interaction and gene × environment interaction for breeding populations in multiple environments. In this study, we used mixed linear model approach and a newly developed mapping software of ***QTXNetwork*** based on GPU parallel computation to identify individual and epistasis loci and their environmental interactions for lint yield and its component traits. We also identified candidate genes related to markers of these target traits. These results and genetic architecture identified in this study could provide more precise and reliable information for marker-assisted selection in crop breeding.

## Materials and Methods

### Plant Materials

Mapping populations representing Upland cotton cultivars and genetic resources were mainly obtained from China and some from foreign countries including the United States of America, Russia, Australia etc (Table S1), which are available from the National Mid-term Genebank of the Institute of Cotton Research, Chinese Academy of Agricultural Sciences (ICR-CAAS) after signing the Material Transfer Agreement (MTA). These cultivars are suitable for association mapping since they have abundant genetic resources. 323 sampled cultivars of Upland cotton were planted and evaluated for lint yield and yield components during 2007 to 2009 in three locations: 1) Anyang of Henan province (annual frost free period = 180–230 days, average active accumulated temperature = 3800–4900°C, annual rainfall = 500–1000 mm) in the Yellow River valley cotton growing region; 2) Kuche of Xinjiang province (annual frost free period = 170–230 days, average active accumulated temperature = 3000–5400°C, annual rainfall = 15–380 mm) in the northwestward cotton growing region; 3) Nanjing of Jiangsu province (annual frost free period = 227–278 days, average active accumulated temperature = 3500–5500°C, annual rainfall = 1000–1600 mm) in the Yangtze River valley cotton growing region. This research is regular cotton regional trial test conducted in farm field of cotton research institute, and not related with protected area of land and protection of wildlife. It does not need specific official permission. We regarded combination of each location and each year as an individual environment, setting a total of nine environments. A randomized complete block design with three replications was employed in the filed trials, and each block was settled with two rows and every row was kept in a plot with 5 m long and 0.7 m wide. The field management utilized conventional field production management techniques adjusted to local practice. Lint yield and three yield components were evaluated: number of bolls per plant, boll weight, and lint percentage.

### DNA Extraction and Marker Analysis

The genomic DNA was extracted from fresh, young leaves of the 323 cultivars using CTAB method. SSR primers sequences including BNL, JESPR, TMB, CIR, HAU, DPL, GH, NAU, MGHES etc. can be available from Cotton Microsatellite Database (CMD, http://www.cottonssr.org). We selected 20 cultivars of large morphological variations from the research natural population to screen for polymorphisms primers. In total, 5,600 SSR primers were chosen to survey the polymorphisms of the 323 cultivars. Only 651 polymorphisms SSR alleles were detected, which were used for association mapping (Table S2).

### Genetic Models and Statistical Methods

Association mapping were performed using the mixed linear model, including environment (*e*) as fixed effects, SSR loci effects (*a, aa*) and loci by environment interaction (*ae, aae*) as random effects. The genetic model for phenotypic value of the *k*-th genotypes in the *h*-th environment (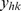) can be expressed by the following mixed linear model,
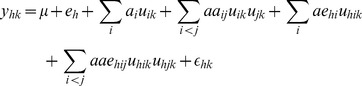
(1)where *µ* is the population mean; 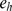 is the fixed effect of the *h*-th environment; 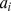 is the *i*-th additive effect with coefficient 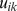; 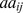 is the *i*-th additive by *j*-th additive epistasis effect with coefficient 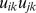; 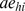 is the *i*-th additive by the *h*-th environment interaction effect with coefficient 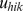; 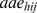 is the 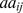 by the *h*-th environment interaction effect with coefficient 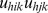; and 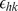 is the random residual effect of the *k*-th breeding line in the *h*-th environment.

The mixed linear model can be presented in matrix notation,
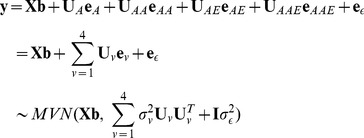
(2)where **y** is an 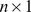 column vector of phenotypic values and 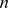 is the sample size of observations; **b** is a column vector of 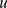 and environments in the experiment; **X** is the known incidence matrix relating to the fixed effects; **U** *_v_* is the known coefficient matrix relating to the *v*-th random vector **e** *_v_*; 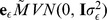 is an 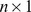 column vector of residual effects.

The phenotypic variance 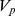 is considered as the sum of additive variance 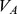, epistasis variance 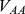, additive by environment interaction variance 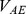, epistasis by environment interaction variance 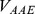, and residual variance 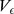.
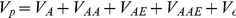
(3)


Heritability is defined as the relative contribution of genetic variance to phenotypic variance with the following estimation model:
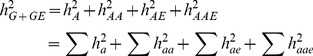
(4)where 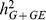 = total heritability; 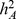 = heritability contributed by sum of individual locus, 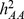 = heritability contributed by sum of pair-wise epistasis, 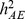 = additive by environment interaction heritability contributed by sum of individual additive by environment interaction effects, 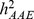 = epistasis by environment interaction heritability contributed by sum of pair-wise epistasis by environment interaction effects; 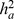 = additive heritability of individual locus, 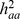 = epistasis heritability of pair-wise loci, 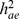 = heritability of additive by environment interaction effect, 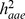 = heritability of epistasis by environment interaction effect.

### Association Mapping

A GPU parallel computing software **QTXNetwork** (http://ibi.zju.edu.cn/software/QTXNetwork/) was used to dissect the genetic architecture of lint yield and its component traits. There were 651 SSR markers used for association mapping for yield traits of 323 representative Upland cotton cultivars in nine different environments. Significant SSR markers were screened and the estimation of fixed effects (*e*) and prediction of random effects (*a, aa, ae*, and *aae*) of loci were obtained.

### Gene Locations Based on the Associated Markers by Reference D Genome

Based on the experiment-wise type I error (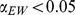) setting by **QTXNetwork**, the SSR loci associated with the fiber yield traits were selected. The primer information was acquired from cotton marker database (http://www.cottonmarker.org/cmd_downloads/ssr_project_data/CMD_PRIMER_ALL.xls). Only one SSR location for each marker on the genome D (ftp://ftp.ncbi.nlm.nih.gov/genbank/genomes/Eukaryotes/plants/Gossypium_raimondii/) was acquired by bowtie method which 2 bases not matched was permitted (bowtie -a -v 2–fr cottonPD -f -1 primerForward.fasta -2 primerReverse.fasta –best –strata bowtieResult_PD.txt). The SSR motifs of the searched SSR sequences on the *D* genome were scanned by MISA. The location between SSR locus and the related gene was decided based the star location of the motif on the genome.

## Results

In total, there were 94 significant SSRs (14 loci with individual effects and 93 loci involved in epistasis interactions) associated with lint yield, 95 SSRs (28 individual loci) associated with boll number, 91 SSRs (21 individual loci) associated with boll weight, and 88 SSRs (19 individual loci) associated with lint percentage. All identified loci associated with lint yield components were involved in epistasis interactions.

**Figure 1:**
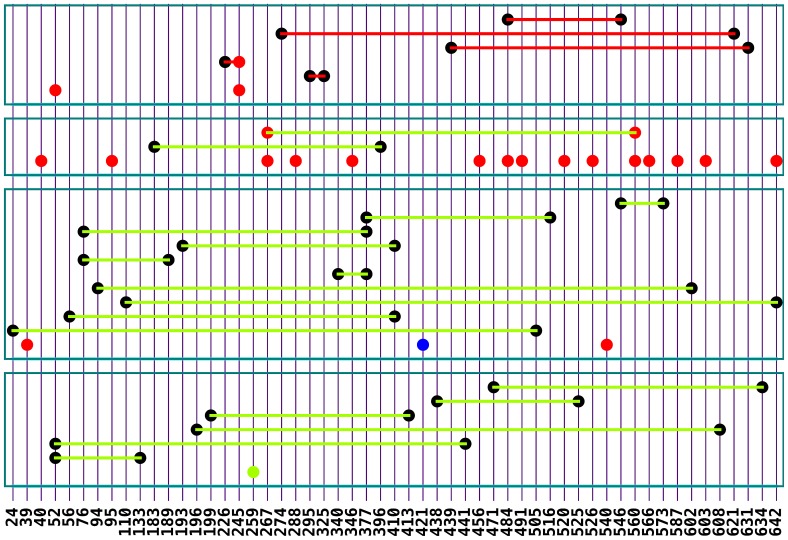
Network plot of highly significant loci detected for yield and yield components. Notes: The bottom axis is the SNP ID for QTS. Red dot = loci with additive effect, green dot = loci with additive × environment interaction effect, blue dot = loci with both additive and environment-specific effects, black dot with a line = loci with epistasis but no individual effect. Red line between two dot = *aa* epistasis, Green line between two dot = *aae* environment-specific epistasis.

As to the lint yield, the total heritability of genotype × environment interaction effects (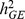
≙38.26%) was mainly contributed due to epistasis × environment interaction (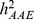
≙34.05%), and was much larger than the total genotype heritability (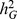
≙10.80%) (Table 1). We listed highly significant genetic effects of loci (−Log_10_ *P*>8.0 and 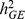
>1.0%, 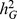
>0.5%) and consistent loci with environment interaction which were stable in a particular ecological area across at least two years for lint yield and three yield traits (Figure 1, Table 2–5). Among the loci associated with lint yield (Table 2), 1 locus (NAU3011-2) and 6 pairs of epistasis loci were highly significant with environmental interaction at Nanjing in 2007 and 2009. It was suggested that improving lint yield could be expected by selecting major-allele genotype *QQ* (NAU3011−197) and *QQ*×*QQ* (MUCS101−330×MGHES18−228, NAU3325−238×TMB1989−255, NAU3608−245×HAU773−170), as well as minor-allele genotype *qq*×*qq* (HAU1794−320×TMB10−375, NAU1362−225×NAU3774−248, and NAU3325−238×HAU423−175) at Nanjing (Table 2).

**Table 1:** Estimated heritability for lint yield and yield components.

Trait	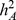 (%)	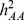 (%)	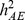 (%)	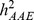 (%)	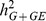 (%)
Lint Yield	0.20	10.6	4.21	34.05	49.06
Boll Number	3.87	9.28	8.89	41.93	63.98
Boll Weight	21.48	22.01	0.63	18.73	62.85
Lint Percentage	6.58	57.78	0.29	7.64	72.29

**Note:** 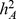 = heritability for additive effects, 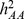 = heritability for additive epistasis effects, 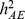 = heritability for additive by environment interaction effects, 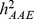 = heritability for additive epistasis by environment effects, 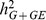 = total heritability.

**Table 2:** Predicted genetic effects with significance and heritability of lint yield for cotton regional trials in three locations and three years.

Locus	Effect	Predict	−Log_10_ *P*	*h^2^* (%)	Candidate Gene
NAU3011−197	*ae*_3_ *y*_1_	1.01	11.32	1.04	IPR000679
	*ae*_3_ *y*_3_	1.56	25.93		
HAU1794−320×TMB10−375	*aae*_3_ *y*_1_	−1.58	26.17	0.96	– × Gorai.004G126800
	*aae*_3_ *y*_3_	−0.99	10.82		
MUCS101−330×MGHES18−228	*aae*_3_ *y*_1_	0.98	10.97	0.65	Cotton_A_29394×IPR001128
	*aae*_3_ *y*_3_	1.06	12.52		
NAU1362−225×NAU3774−248	*aae*_3_ *y*_1_	−0.84	8.05	0.79	IPR001764×IPR002913
	*aae*_3_ *y*_2_	−1.15	14.61		
	*aae*_3_ *y*_3_	−1.04	12.03		
NAU3325−238×HAU423−175	*aae*_3_ *y*_1_	−1.03	11.93	0.72	IPR000778×IPR001461
	*aae*_3_ *y*_3_	−1.25	17.12		
NAU3325−238×TMB1989−255	*aae*_3_ *y*_1_	1.44	21.74	1.00	IPR000778×Cotton_A_24169
	*aae*_3_ *y*_3_	1.15	14.23		
NAU3608−245×HAU773−170	*aae*_3_ *y*_1_	1.10	13.26	0.73	IPR000719×IPR007087
	*aae*_3_ *y*_3_	0.89	8.99		

**Note:** *ae*_3_ *y*_1_ = additive by environment interaction effect at Nanjing in 2007; *ae*_3_ *y*_3_ = additive by environment interaction effect at Nanjing in 2009; *aae*_3_ *y*_1_ = epistasis by environment interaction effect at Nanjing in 2007; *aae*_3_ *y*_2_ = epistasis by environment interaction effect at Nanjing in 2008; *aae*_3_ *y*_3_ = epistasis by environment interaction effect at Nanjing in 2009; −Log_10_ *P* = minus log_10_ (*P-*value)**,** *h* ^2^ (%) = heritability (%).

**Table 3:** Predicted genetic effects with significance and heritability of boll number for cotton regional trials in three locations and three years.

Locus	Effect	Predict	−Log_10_ *P*	*h^2^* (%)	Candidate Gene
HAU1385−150	*a*	0.3	42.29	0.68	IPR003245
MGHES41−330	*a*	−0.27	34.72	0.55	IPR000297
NAU1102−241	*a*	−0.28	37.61	0.6	IPR003329
	*ae*_3_ *y*_1_	−0.38	8.35	0.63	
	*ae*_3_ *y*_2_	−0.46	11.99		
	*ae*_3_ *y*_3_	−0.52	14.8		
GH111−245×NAU874−215	*aae*_3_ *y*_1_	0.37	8.25	0.37	– ×IPR001128
	*aae*_3_ *y*_3_	0.39	8.93		
GH111−245×NAU5433−330	*aae*_3_ *y*_1_	0.55	16.75	0.96	– ×IPR001865
	*aae*_3_ *y*_3_	0.84	37.43		
HAU1029−197×NAU1125−243	*aae*_1_ *y*_2_	0.47	12.16	1.59	IPR001012×IPR016196
	*aae*_1_ *y*_3_	0.55	16.85		
	*aae*_3_ *y*_1_	0.93	46.13		
	*aae*_3_ *y*_3_	0.63	21.32		
HAU1969−375×JESPR42−128	*aae*_3_ *y*_1_	1.08	60.86	2.43	IPR000181×Gorai.009G322400
	*aae*_3_ *y*_3_	1.27	83.72		
HAU1639−320×NAU5099−233	*aae*_3_ *y*_1_	0.38	8.55	0.53	IPR000217×IPR001214
	*aae*_3_ *y*_3_	0.5	14.18		
NAU2715−184×JESPR42−128	*aae*_3_ *y*_2_	−0.63	22.12	0.65	IPR004022×Gorai.009G322400
	*aae*_3_ *y*_3_	−0.38	8.43		
NAU2873−352×NAU5433−330	*aae*_3_ *y*_1_	−0.50	13.93	0.77	IPR003311×IPR001865
	*aae*_3_ *y*_3_	−0.77	32.56		
NAU4042−175×GH111−245	*aae*_3_ *y*_1_	0.47	12.53	0.64	Cotton_A_04013×–
	*aae*_3_ *y*_3_	0.68	25.62		
STV61-131×NAU3305−155	*aae*_3_ *y*_1_	−0.91	43.35	1.49	DUF1685×IPR002913
	*aae*_3_ *y*_2_	−0.65	22.48		
TMB1181−220×NAU3563−149	*aae*_1_ *y*_1_	0.6	19.26	1.25	Gorai.002G088500×IPR003311
	*aae*_1_ *y*_2_	0.42	9.91		

**Note:** *a = *additive effect; *ae*_3_ *y*_1_, *ae*_3_ *y*_3_, *aae*_3_ *y*_1_, *aae*_3_ *y*_2_, *aae*_3_ *y*_3_ as defined in **Table 2**; *ae*_3_ *y*_2_ = additive by environment interaction effect at Nanjing in 2008, *aae*_1_ *y*_1_ = epistasis by environment interaction effect at Anyang in 2007, *aae*_1_ *y*_2_ = epistasis by environment interaction effect at Anyang in 2008, *aae*_1_ *y*_3_ = epistasis by environment interaction effect at Anyang in 2009; −Log_10_ *P* = minus log_10_ (*P-*value)**,** *h* ^2^ (%) = heritability (%).

**Table 4:** Predicted genetic effects with significance and heritability of boll weight for cotton regional trials in three locations and three years.

Locus	Effect	Predict	−Log_10_ *P*	*h^2^*(%)	Candidate Gene
NAU3744−200	*a*	−0.07	66.02	1.29	IPR020828
TMB312−212	*a*	−0.06	54.75	1.07	IPR011335
TMB1296−230	*a*	0.07	60.15	1.18	IPR002591
BNL1313-180	*a*	0.08	95.32	1.89	–
NAU3588−315	*a*	−0.15	299.91	6.14	–
GH132−180	*a*	−0.06	47.17	0.92	IPR001611
JESPR274−129	*a*	−0.06	43.33	0.84	–
JESPR101−122	*a*	0.05	42.82	0.83	IPR004813
NAU3305−155	*a*	−0.05	42.63	0.83	IPR002913
NAU5099−280	*a*	0.05	42.79	0.83	IPR001214
TMB1963−243	*a*	−0.05	37.99	0.73	–
HAU1385−155	*a*	0.05	36.04	0.7	IPR003245
BNL3033−175	*a*	−0.05	33.68	0.65	IPR008540
NAU2931−250	*a*	−0.04	27.91	0.53	IPR001841
NAU1163-160	*a*	−0.04	26.63	0.51	–
TMB312−212×GH132−180	*aae*_2_ *y*_1_	−0.08	11.95	0.35	IPR011335×IPR001611
	*aae*_2_ *y*_2_	−0.07	8.79		
NAU3013−245×NAU5163−216	*aae*_3_ *y*_1_	−0.09	12.53	0.66	Cotton_A_34510×IPR002917
	*aae*_3_ *y*_3_	−0.08	10.01		

**Note:** *a = *additive effect, *aa* = additive by additive epistasis effect; *aae*_2_ *y*_1_ = epistasis by environment interaction effect at Kuche in 2007; *aae*_2_ *y*_2_ = epistasis by environment interaction effect at Kuche in 2008; *aae*_3_ *y*_1_ = epistasis by environment interaction effect at Nanjing in 2007. *aae*_3_ *y*_3_ = epistasis by environment interaction effect at Nanjing in 2009; −Log_10_ *P* = minus log_10_ (*P-*value)**,** *h* ^2^ (%) = heritability (%).

**Table 5:** Predicted genetic effects with significance and heritability of lint percentage for cotton regional trials in three locations and three years.

Loci	Effect	Predict	−Log_10_ *P*	*h* ^2^ (%)	Candidate Gene
NAU3325−238	*a*	−0.44	164.11	2.47	IPR000778
NAU3519−200	*a*	−0.23	46	0.67	IPR000719
NAU3519−220× TMB1268−157	*aa*	0.29	71.31	1.04	IPR000719× IPR002048
HAU1185−174×TMB1791−217	*aa*	−0.21	38.63	0.55	IPR000194 ×IPR000209
TMB1638−189×DPL513−320	*aa*	0.21	37.87	0.54	IPR002109 ×–
NAU3110−292×NAU2862−248	*aa*	−0.20	36.04	0.52	IPR003329 ×IPR001841
JESPR101−122×BNL1231−228	*aa*	−0.20	35.61	0.51	IPR004813 × –

**Note:** **Genetic effect:** *a* = additive effect, *aa* = additive by additive epistasis effect; −Log_10_ *P* = minus log_10_ (*P-*value), *h* ^2^ (%) = heritability (%).

For boll number trait, the heritability of epistasis × environment interaction effects (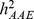
≙41.93%) was larger than other trait heritability (Table 1), which indicated that boll number was mainly controlled by environment-specific epistasis effects. 3 loci (HAU1385-3, NAU1102-3, MGHES41-6) with additive heritability were found to be more than 0.5%, and 10 pair epistasis loci with environment-specific epistasis heritability showed more than 1.0% genetic effects (Figure 1, Table 3), indicating that the improvement of boll number in all 9 environments could be predictable (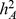
≙1.83%) by selecting genotype *QQ* for HAU1385−150, and *qq* for other 2 loci (MGHES41−333, NAU1102−241). Further improving boll number could be expected (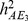
≙11.31%) through selecting HAU1029−197 × NAU1125−243 in both Anyang and Nanjing areas and other 9 pair epistasis loci only in Nanjing area, among these 7 epistasis loci with genotype of *QQ*×*QQ* showed positive effects, and 3 pair epistasis loci with genotype of *qq*×*qq* showed negative effects. The total heritability (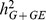
≙ 62.85%) of boll weight was mostly due to additive effects (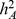
≙21.48%), epistasis effects (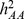
≙22.01%) and environment-specific additive effects (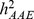
≙18.73%). It was suggested that most genetic effects were fairly stable across environments (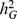
≙43.49%) for boll weight. There were 15 loci with heritability of additive effect larger than 0.5% and 2 pair epistasis loci with heritability of environment-specific epistasis effects larger than 1.0% for boll weight (Figure 1, Table 4). With regard to little impact of environmental factors on boll weight, relevant robust loci could be selected for improvement of lint yield across different ecological areas.

For lint percentage, the total heritability (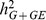
≙72.29%) was mostly attributed to epistasis effects (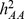
≙57.78%). Thus, it was concluded that lint percentage was very stable across various environments and mainly controlled by epistasis effects. Compared with lint yield and other yield components, most genetic effects of individual loci and epistasis loci explained a small proportion of total phenotypic variance for lint percentage. Only 2 loci with additive effects and 5 pair epistasis loci with epistasis effects had heritability larger than 0.5% (Figure 1 and Table 5). It was apparent that lint percentage was mostly controlled by many loci with smaller effects as compared with lint yield and other component traits. In this population, further genetic gain could be projected (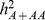
≙ 6.3%) based on selecting 2 individual loci with additive effects (NAU3325−238, NAU3519−200) and 5 pair loci with epistasis effects for increasing lint percentage across different environments.

It has been observed that some loci were associated with more than one trait. 4 loci (DPL910-120, HAU639-175, NAU1155-205, NAU4042−175) were found to be associated with all of the four traits, 22 loci were detected to be associated with three of the four traits, and 61 loci were associated with two of the four traits. Generally, these common loci made it possible for us to simultaneously improve multiple traits via breeding programs. However, 41 loci controlling lint yield were not detected in other component traits, which suggested that lint yield, as the result of complex biosynthesis pathways, may comprise some unknown components affected by polygenes.

It had been observed that epistasis is the genetic base of lint yield and yield components. In the results, most of the loci with individual effects were involved in epistasis interactions for lint yield and yield components. Altogether 293 digenic loci with epistasis effect (*aa*) and/or epistasis × environment interaction effect (*aae*) were identified to be associated with lint yield. There were 193 digenic epistasis interactions with their total heritability (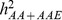
≙ 30.71%) of epistasis effects and environment-specific epistasis effects involved in the loci without individual effects. We also observed this phenomenon in yield components. It was shown that many loci, even without controlling yield and yield components on their own, could affect the traits in combination with other loci.

It was observed that many loci could interact with several other loci for controlling phenotypic variation of lint yield and its component traits. For example, NAU2126-195 could affect lint yield in combination with other 4 loci (HAU773−170, NAU3377-180, TMB10−375, GH501-265), respectively. Magnitude of epistasis effects of them differed from each other. NAU2126-195 may be regarded as core locus of epistasis interaction jointly with other casual genes for controlling target traits. This phenomenon might indicate that higher order interaction could also exist for association with lint yield and its components.

## Discussion

Association mapping is a powerful method to detect loci underlying complex traits and more efficient compared to linkage analysis, because it exploited abundant genetic variation in diverse genetic backgrounds [15]. However, in most of the current GWAS, the epistasis and environmental interaction effects were not detectable due to absence of appropriate statistical methods and a heavy computational burden [16]–[18]. The models without epistasis and environmental interaction for GWAS may cause some problems: 1) It may result in biased estimation of effects on loci and decrease the precision and power of loci detection [18]–[19]; 2) The heritability of complex traits would be missed in the condition of reduced models [20]–[21].

Our proposed methodology is based on mixed linear model including epistasis and environmental interaction, but it can also be used for reduced models ignoring epistasis and environmental interaction. We also performed GWAS of various reduced models for lint yield and yield components. As compared with the full model (Table 1), the total heritability of lint yield and yield components decreased in the additive model (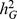
≙18.35% for lint yield, 6.53% for boll number, 28.65% for boll weight, and 22.95% for lint percentage), and also in the additive with environmental interaction model (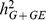
≙17.31% for lint yield, 23.76% for boll number, 32.25% for boll weight, and 25.08% for lint percentage). The results also showed steep decrease in numbers and effects of loci associated lint yield and yield components between reduced models and full model. This may explain the reasons for missing heritability reported recently [21].

Because of low LD associated with higher frequencies of cross-pollination, cotton species are amenable for association mapping of agronomic traits with a relatively large numbers of markers. Previous studies involving nearly 1000 polymorphic markers would ensure the coverage of whole cotton genome [14]. A total of 651 SSR markers and 323 Upland cotton cultivars under 9 environments in this study can significantly improve the power of association mapping. However, with the rapid development of next generation sequencing, a substantial number of single nucleotide polymorphisms (SNPs) will facilitate the accurate and fine association analysis.

It is shown by the results of GWAS that lint yield, boll number and lint percentage are inherited in a mainly epistasis manner, whereas boll weight is predominately controlled by both additive and epistasis effects. In addition, lint yield and boll number are particularly susceptible to a broad range of environments, as compared to boll weight and lint percentage, which has also been observed in previous studies [5], [22]–[23]. Both lint yield and component traits were complex traits controlled by many genes and gene-network cascades interacting with each other and also with the environment factors (Figure 1). Many loci (ten or more in the present study) associated with lint yield, boll number, and boll weight showed much larger effects and heritability (additive or epistasis effects), while few loci associated with lint percentage could have large effects. This was indicated that improving cotton lint yield could be difficult through increasing lint percentage. All these information obtained from GWAS could provide useful reference for MAS in cotton breeding programs.

Although the effects of many loci appeared to be consistent across various environments, the magnitude of effects and even direction of loci may vary depending on environmental factors because of interactions between loci and environment [3], [18], [24]. We can classify loci identified in the present study into three types. The first category of loci is called constituted loci with large major effects (*a, aa*) and can be consistently detected across different environments. This type of loci is very useful for improving cotton traits across different ecological locations. Another type of loci is called environment-specific loci with only environment-special effects identified in a special ecological region but stable in different years. For example, TMB312−212 × GH132-180 was stably found in the Northwestward region and NAU3013−245 × NAU5163−216 was stably scanned in the Yangtze River valley region. This type of loci could be used for maker-assisted breeding in a particular ecological area. The third type of loci is called environment-sensitive loci that could not be consistently detected in various grown regions. This kind of loci could only be used in specific conditions.

SSRs widely used in major crop were highly reliable, polymorphic, simple and cheap. The SSRs associated with nearby genes were extremely useful for MAS. In the present study, we successfully detected 251 significant loci associated with lint yield and its three components, including 69 loci with individual effects. Most of these significant loci generally had small impacts on cotton breeding due to small effects and easily affected by environment [23]. In order to develop effective MAS schemes, reliable and elite loci associated with lint yield and yield components have been screened. These loci, which consist of constituted loci and environment-specific loci, are reliable to predict corresponding phenotype. Constituted loci (such as NAU3588−315 and NAU3325−238) can be applied for MAS in various cotton-growing region, whereas application of environment-specific loci (examples are NAU1102−241 and NAU1362−225 × NAU3774−248) are limited to specific cotton-growing region. Both of them have substantial potential to improve the efficiency and precision of conventional cotton breeding.

With the information from Cotton Microsatellite Database (CMD) and published report, several markers identified in our present study had been located within or nearby the reported QTL in previous linkage analysis and association mapping [1]–[8], [14]. For example, Zhang et al [14] independently detected QTLs called qLP-A1-1 (between JESPR101 and BNL3590) using linkage method and a locus called JESPR101 using association method, which affected lint percentage. We also identified this marker (−Log_10_ *P*-value = 15.09) associated with the same (Table 5). In addition, most of these consistent loci were involved in interaction in our present results (examples are NAU5433−330 for numbers of boll in Table 3). It indicated that these loci were stably transferred and can be further used in MAS.

The traditional QTL was mainly located between two markers with the scale of cM (centi-Mogan). Based on the physical mapping of reference genome, the fine mapping of the markers and the genes related to the fiber yield traits become possible. In this study, candidate gene locations of yield fiber traits were identified based on the associated SSR markers analyzed by the ***QTXNetwork***. For the lint yield, one major-allele genotype *QQ* (NAU3011−197) related to zinc finger (GATA-type) gene (IPR000679) was identified. 3 epistasis loci with positive effects (*QQ*×*QQ* genotype) could be related to the interactions with the genes of cytochrome P450 (IPR001128), Cytochrome b245 (IPR000778), zinc finger with *C2H2*-type (IPR007087), and protein kinase (IPR000719) etc. 3 minor-allele with negative effects (*qq*×*qq* genotype) was linked to the interactions with the genes of glycoside hydrolase (IPR001764), lipid-binding *START* (IPR002913), and peptidase aspartic (IPR021109) at Nanjing areas (Table 2). It seems that the genes catalyzed the oxidation of organic substances will have positive effects on cotton lint yield, but the decompose genes of glycoside, lipid and protein maybe have negative effects for lint yield.

For boll number, there were 3 additive SSR loci (HAU1385-150, NAU1102−241, MGHES41−333) related to genes of peptidyl-prolyl cis-trans isomerases (PPIase, IPR000297), plastocyanin-like (IPR003245), and acylneuraminate cytidylyltransferase (IPR003329). 7 epistasis loci with positive effects for boll number (*QQ*×*QQ* genotype) might be related to gene interactions of cytochrome *P450* (IPR001128), *UBX* domain-containing protein (IPR001012), tubulin (IPR000217), ribosomal protein (IPR001865), major facilitator superfamily (IPR016196), formylmethionine deformylase (IPR000181), and SET domain of SUVH4 (IPR001214) etc. There were 3 epistasis loci with negative effects (*qq*×*qq* genotype) related to gene interactions of *DDT* domain superfamily (IPR004022), *AUX/IAA* protein (IPR003311), ribosomal protein (IPR001865), protein of unknown function (DUF1685), lipid-binding *START* (IPR002913) etc. It seems that the gene interactions for the metabolism and transport of cysteine, prolyl and formylmethionine could increase the boll number. But the regulation of the gene interactions of the lipid-binding DNA binding and auxin-binding protein could also affect the boll number.

For boll weight, there were 15 additive SSR loci with positive effects relate to following genes: Type I phosphodiesterase (IPR002591), *SET* domain (IPR001214) of *SUVH4*, plastocyanin-like (IPR003245), oligopeptide transporter (OPT) superfamily (IPR004813). While the genes could have negative effects, such as glyceraldehyde 3-phosphate dehydrogenase (IPR020828), restriction endonuclease (IPR011335), leucine-rich repeat (IPR001611), STAR-related lipid-transfer (*START*) domain (IPR002913), brassinosteroid signalling positive regulator (*BZR1*) family protein(IPR008540), and RING-type zinc finger (IPR001841), respectively. Except the main additive effects, there are also 2 epistasis loci with negative effects (*qq*×*qq* genotype) which maybe resulted from the interactions of leucine-rich repeat (IPR001611), GTP-binding protein (IPR002917) etc. It was inferred that boll weight is mainly controlled by the genes with the phosphorus, lipid and signalling transport, which may be further regulated by the zinc finger, leucine-rich repeat, and the methylation protein.

For lint percentage, 2 SSR loci with negative additive effects could be related to protein kinase (IPR000719) and cytochrome b245 (IPR000778), respectively. There were 2 epistasis loci with positive effects (*QQ*×*QQ* genotype) related to the interactions of the genes of protein kinase (IPR000719), calcium-binding EF-hand (IPR002048), glutaredoxin (IPR002109). Three epistasis loci with negative effects (*qq*×*qq* genotype) relate to the interactions of the genes of peptidase S8/S53 (IPR000209), zinc finger with RING-type (IPR001841), and oligopeptide transporter OPT superfamily (IPR004813).

## Supporting Information

Table S1 **Variety germplasm of Upland cotton used in association mapping for fiber traits.** Note: New lines were new breeding lines and genetic stocks bred by Institute of Cotton Research of Chinese Academy of Agricultural Sciences (ICR, CAAS) in recent years.
(DOCX)

Table S2 **651 primer alleles that were polymorphic in the tested population.**
(XLS)
