# Supplementary material for: Association Mapping for Epistasis and Environmental Interaction of Yield Traits in 323 Cotton Cultivars under 9 Different Environments
Source: PLoS One. 2014 May 8;9(5):e95882. doi: 10.1371/journal.pone.0095882 (PMC4014473; doi:10.1371/journal.pone.0095882)
Supplement: Table S1 — Variety germplasm of Upland cotton used in association mapping for fiber traits. Note: New lines were new breeding lines and genetic stocks bred by Institute of Cotton Research of Chinese Academy of Agricultural Sciences (ICR, CAAS) in recent years. (DOCX) [file pone.0095882.s001.docx]

Table S1. Variety germplasm of Upland cotton used in association mapping for fiber traits

| Serial No. | Reg. No. | Germplasm Name | Geographical Origin |
| --- | --- | --- | --- |
| V9 | 110313 | HuaiMian4Hao | Anhui, China |
| V17 | 110644 | WanJiu828 | Anhui, China |
| V62 | 110737 | XiaoXianDaLing | Anhui, China |
| V324 | 112240 | SuMian9108 | Anhui, China |
| V266 | 112572 | WangJiangChangRongMian | Anhui, China |
| V152 | 110761 | 86-1 (72-100) | BeiJing, China |
| V132 | 111550 | ZhongZhiBD13 | BeiJing, China |
| V133 | 111552 | ZhongZhiBD27 | BeiJing, China |
| V134 | 111553 | ZhongZhiBD89 | BeiJing, China |
| V210 | 112795 | GK20 | BeiJing, China |
| V116 | 112796 | GK22 | BeiJing, China |
| V85 | 111027 | NaShangQuDaHua | Guizhou, China |
| V55 | 110070 | JiHan2HaoXuanXi | Hebei, China |
| V4 | 110083 | Bao6722 | Hebei, China |
| V284 | 110097 | 353DaLingDi3Xi | Hebei, China |
| V278 | 110730 | HanDanChangRong | Hebei, China |
| V286 | 110733 | ChengDingDaLing | Hebei, China |
| V3 | 110810 | Bao6716 | Hebei, China |
| V41 | 110861 | Jimian11 (CK) | Hebei, China |
| V86 | 110862 | JiMian12Hao (Handan177) | Hebei, China |
| V93 | 110938 | XingTai6 | Hebei, China |
| V227 | 111018 | HanDan568 | Hebei, China |
| V96 | 111081 | XingTai79-11 | Hebei, China |
| V21 | 111346 | Han8901 | Hebei, China |
| V123 | 111373 | Han8944 | Hebei, China |
| V22 | 111381 | Han8959 | Hebei, China |
| V326 | 112019 | JiYuan12-13 | Hebei, China |
| V260 | 112022 | JiYuan55 (91Han14) | Hebei, China |
| V279 | 112023 | JiHaiLu6 (91Han6) | Hebei, China |
| V24 | 112043 | Ji91-31 | Hebei, China |
| V252 | 112046 | Ji91-33 | Hebei, China |
| V237 | 112123 | Ji85-3 | Hebei, China |
| V235 | 112126 | Ji91-12 | Hebei, China |
| V301 | 112128 | Ji91-18 | Hebei, China |
| V231 | 112132 | Ji91-22 | Hebei, China |
| V238 | 112135 | Ji91-32 | Hebei, China |
| V194 | 112144 | JiA-1-7 (33Xi) | Hebei, China |
| V120 | 112145 | JiA-7-8 (33Xi) | Hebei, China |
| V45 | 112194 | ShiYuan638 | Hebei, China |
| V257 | 112233 | JiZi123 (Jimian25) | Hebei, China |
| V117 | 112714 | Han109 | Hebei, China |
| V247 | 112714 | HanDan109 | Hebei, China |
| V268 | 112733 | Ji668 | Hebei, China |
| V270 | 112751 | HanDan333 | Hebei, China |
| V217 | 112773 | SGKShiXuan321 | Hebei, China |
| V243 | 113561 | GK99-1 | Hebei, China |
| V81 | 114008 | JiFeng197 | Hebei, China |
| V5 | 110033 | ZhangDe184 | Henan, China |
| V144 | 110323 | ZhongYuan9114 | Henan, China |
| V276 | 110477 | ZhengZhouZhangRongMian | Henan, China |
| V90 | 110508 | Yu284 | Henan, China |
| V149 | 110544 | Zhong521 | Henan, China |
| V80 | 110722 | DaTaoMian | Henan, China |
| V58 | 110728 | NeiHuangDaZi | Henan, China |
| V68 | 110729 | MeiFuDaLing | Henan, China |
| V295 | 110839 | ZhongMianSuo12 | Henan, China |
| V103 | 110855 | Zhong2108 | Henan, China |
| V175 | 110856 | ZhongMianSuo17Hao (Zhong117) | Henan, China |
| V176 | 110919 | ZhongMianSuo19Hao (Zhong7886) | Henan, China |
| V92 | 110921 | Zhong85271 | Henan, China |
| V293 | 110959 | XinXiang89S-210 | Henan, China |
| V70 | 110961 | XinDaLing | Henan, China |
| V203 | 111006 | Zhong1276 | Henan, China |
| V229 | 111037 | ZhongZi9103 | Henan, China |
| V135 | 111117 | ZhongZi640 | Henan, China |
| V226 | 111180 | Zhong932906 | Henan, China |
| V91 | 111184 | Zhong5913-2 | Henan, China |
| V228 | 111210 | ZhongZi9102 | Henan, China |
| V19 | 111225 | ChangRongMuZiMian | Henan, China |
| V163 | 111336 | Zhong89-1 | Henan, China |
| V261 | 111395 | RTBaiXu | Henan, China |
| V143 | 111421 | ZhongYuan911 | Henan, China |
| V150 | 111422 | ZhongYuan9112 | Henan, China |
| V146 | 111424 | ZhongYuan9116 | Henan, China |
| V145 | 111425 | ZhongYuan9115 | Henan, China |
| V147 | 111431 | ZhongYuanHAS-1 | Henan, China |
| V259 | 111434 | ZhongYuanHST1 | Henan, China |
| V290 | 111895 | ZhongMianSuo16 | Henan, China |
| V211 | 111900 | YuMian９Hao (Yuzao1109) | Henan, China |
| V251 | 111944 | Zhong07 | Henan, China |
| V130 | 111950 | Zhong870203 | Henan, China |
| V131 | 111961 | Zhong961716 | Henan, China |
| V151 | 112196 | YuMian2067 | Henan, China |
| V212 | 112241 | ZhongMianSuo27 | Henan, China |
| V215 | 112247 | ZhongMianSuo35 | Henan, China |
| V265 | 112570 | YuMian19 | Henan, China |
| V292 | 112686 | ZhongMianSuo41 (SGK9708) | Henan, China |
| V104 | 112690 | ZhongJi926 | Henan, China |
| V206 | 112698 | ZhongR03 | Henan, China |
| V287 | 112726 | XinYan96-48 | Henan, China |
| V118 | 112746 | ZhongMianSuo32 | Henan, China |
| V124 | 112782 | KangHuangWei164 | Henan, China |
| V289 | 112798 | ZhongZi9196 (Jiguang) | Henan, China |
| V306 | 112799 | Zhong2220 | Henan, China |
| V102 | 113229 | ZhengKang01-505 | Henan, China |
| V299 | 113693 | ZhongMianSuo50 | Henan, China |
| V61 | 113697 | ZhongZi4480 | Henan, China |
| V298 | 113939 | ZhongMianSuo49 | Henan, China |
| V172 | 113943 | ZhongZi04184 | Henan, China |
| V321 | 114019 | Zhong2201 | Henan, China |
| V325 | 114152 | ZhongZi10Hao | Henan, China |
| V312 | 114483 | ZhongG5 | Henan, China |
| V37 | 130293 | ZaoShuChangRong7 | Henan, China |
| V106 | 131404 | CZA(70)33 | Henan, China |
| V262 | 131624 | MSCO-11 | Henan, China |
| V282 | 131625 | MSCO-12 | Henan, China |
| V59 | 131676 | NongJiu | Henan, China |
| V153 | 140135 | ZiSeMeiMian | Henan, China |
| V288 | 140152 | YuMian2Hao (YuWu302) | Henan, China |
| V38 | 140338 | YaHuang9103 | Henan, China |
| V323 | 140444 | LangHuangF10 (ZongXu) | Henan, China |
| V51 | New lines | ZhongARR40682 | Henan, China |
| V52 | New lines | ZhongARNnXu | Henan, China |
| V72 | New lines | ZhongＲ773-72 | Henan, China |
| V73 | New lines | ZhongARR40681 | Henan, China |
| V74 | New lines | ZhongARR40683 | Henan, China |
| V75 | New lines | ZhongR773-75 | Henan, China |
| V76 | New lines | ZhongArc-76 | Henan, China |
| V77 | New lines | ZhongAR683-77 | Henan, China |
| V78 | New lines | RTN78 | Henan, China |
| V79 | New lines | RTN79 | Henan, China |
| V105 | New lines | ZhongArc-105 | Henan, China |
| V119 | New lines | ZhongAR40772 | Henan, China |
| V177 | New lines | Su08B2-177 | Henan, China |
| V185 | New lines | ZhongArc-185 | Henan, China |
| V187 | New lines | RNX187 | Henan, China |
| V188 | New lines | RNX188 | Henan, China |
| V189 | New lines | RNX189 | Henan, China |
| V190 | New lines | RNX190 | Henan, China |
| V202 | New lines | Yu17-202 | Henan, China |
| V204 | New lines | Zhong31-204 | Henan, China |
| V205 | New lines | Zhong4612YaH | Henan, China |
| V221 | New lines | Zhong9708-221 | Henan, China |
| V222 | New lines | Zhong9708-222 | Henan, China |
| V223 | New lines | TM1-IPR | Henan, China |
| V307 | New lines | Zhong507145 | Henan, China |
| V308 | New lines | ZhongArc-308 | Henan, China |
| V309 | New lines | ZhongR773-309 | Henan, China |
| V310 | New lines | ZhongR773-310 | Henan, China |
| V311 | New lines | ZhongAR-RNX311 | Henan, China |
| V313 | New lines | ZhongAR-RNX313 | Henan, China |
| V314 | New lines | ZhongR773-314 | Henan, China |
| V315 | New lines | ZhongArc-315 | Henan, China |
| V316 | New lines | ZhongAR681-316 | Henan, China |
| V317 | New lines | 99633 | Henan, China |
| V320 | New lines | Ari-320 | Henan, China |
| V322 | New lines | HongTao | Henan, China |
| V327 | New lines | Ari-327 | Henan, China |
| V329 | New lines | ZhongR773-329 | Henan, China |
| V6 | 110639 | ChangRong67-12 | Hubei, China |
| V83 | 110746 | HuBeiSongZiDaLing | Hubei, China |
| V71 | 110747 | XinZhouDaLing | Hubei, China |
| V26 | 111664 | Jing55263 | Hubei, China |
| V20 | 111709 | E408 | Hubei, China |
| V42 | 112272 | Jing55168 | Hubei, China |
| V23 | 112527 | HuaZhong91-0102 | Hubei, China |
| V267 | 112705 | EKangMian9Hao | Hubei, China |
| V35 | 110235 | XiangMian2Hao | Hunan, China |
| V28 | 110743 | LiXianDaTao | Hunan, China |
| V67 | 110744 | DaLingMian69Hao | Hunan, China |
| V13 | 110286 | ShaMaoChangTao | Jiangsu, China |
| V272 | 110381 | 601ChangRongMian | Jiangsu, China |
| V218 | 110614 | SiMian2Hao | Jiangsu, China |
| V14 | 110646 | SiChang2Xi | Jiangsu, China |
| V69 | 110731 | QiFengDaLing | Jiangsu, China |
| V56 | 110738 | JiangSuDaTao | Jiangsu, China |
| V285 | 110740 | XuShiDaTao | Jiangsu, China |
| V98 | 111022 | XuZhouBanBanMian | Jiangsu, China |
| V277 | 111086 | SuMian2Hao (Xuzhou553) | Jiangsu, China |
| V15 | 111398 | Su7036YuanYuan | Jiangsu, China |
| V200 | 111401 | SuYuan7235 | Jiangsu, China |
| V99 | 111409 | YanCheng1115 | Jiangsu, China |
| V47 | 112189 | SuXu137 (Zaoshu) | Jiangsu, China |
| V48 | 112190 | SuXu138 (Zaoshu) | Jiangsu, China |
| V121 | 112581 | JCG59 | Jiangsu, China |
| V122 | 112593 | JCG94 | Jiangsu, China |
| V148 | 112697 | ChangKangMian | Jiangsu, China |
| V258 | 112734 | XuZhou261 | Jiangsu, China |
| V269 | 112734 | XuZhou261 | Jiangsu, China |
| V95 | 112736 | Si168 | Jiangsu, China |
| V97 | 112755 | XuZhou244 | Jiangsu, China |
| V198 | 113189 | SuQ1 | Jiangsu, China |
| V199 | 113190 | SuTKH-1 | Jiangsu, China |
| V16 | 113368 | SuNong6Hao | Jiangsu, China |
| V319 | 113675 | SuYuan04-129 | Jiangsu, China |
| V49 | 113676 | J02-247 | Jiangsu, China |
| V50 | 113677 | J02-508 | Jiangsu, China |
| V318 | 113691 | SuYuan04-162 | Jiangsu, China |
| V65 | 130452 | DaLingFuZiMian | Jiangsu, China |
| V281 | 110600 | GaoYiMian | Jiangxi, China |
| V7 | 110176 | ZhaoYang70Hao | Liaoning, China |
| V154 | 111762 | LiaoMian-5Hao | Liaoning, China |
| V18 | 111772 | Liao61107 | Liaoning, China |
| V10 | 111783 | Jin444 | Liaoning, China |
| V246 | 112337 | Liao96-63-70 | Liaoning, China |
| V241 | 112341 | Liao96-23-30 | Liaoning, China |
| V248 | 112372 | Liao823-834-23 | Liaoning, China |
| V138 | 113790 | LiaoMian16 | Liaoning, China |
| V139 | 113791 | LiaoMian17 | Liaoning, China |
| V140 | 113792 | LiaoMian18 | Liaoning, China |
| V296 | 113793 | LiaoMian19 | Liaoning, China |
| V137 | 113811 | Liao4853 | Liaoning, China |
| V136 | 113842 | Liao4835 | Liaoning, China |
| V274 | 110131 | DaZeMian | Shandong, China |
| V94 | 110486 | ShanNong3Hao | Shandong, China |
| V36 | 110671 | YangFen31Hao | Shandong, China |
| V89 | 110942 | LuMian11Hao (3389) | Shandong, China |
| V88 | 110947 | LinQing201 | Shandong, China |
| V264 | 112552 | Lu458 | Shandong, China |
| V82 | 112952 | Lu21 | Shandong, China |
| V273 | 112952 | LuMianYan21(Lu1138) | Shandong, China |
| V44 | 113219 | ShanNong6Hao | Shandong, China |
| V195 | 113756 | LuNong9648 | Shandong, China |
| V8 | 111620 | Hu749513 | Shanghai, China |
| V201 | 110182 | ShenMian5Hao | ShanXi, China |
| V127 | 111181 | Shan3184 | ShanXi, China |
| V234 | 111274 | Tai83158 | ShanXi, China |
| V236 | 111493 | JinKang157 | ShanXi, China |
| V232 | 111495 | Jin90Kang282 | ShanXi, China |
| V128 | 111937 | Shan960329-2Yuan3 | ShanXi, China |
| V125 | 112106 | Shan2747 | ShanXi, China |
| V126 | 112108 | Shan2800 | ShanXi, China |
| V100 | 112295 | Yun3060 | ShanXi, China |
| V129 | 112322 | Yun92Kang124 | ShanXi, China |
| V242 | 112324 | Yun93Kang354 | ShanXi, China |
| V244 | 112325 | Yun93Kang393 | ShanXi, China |
| V101 | 112331 | Yun92A-260 | ShanXi, China |
| V275 | 112550 | QinYuan4Hao (Qinyuan91406) | ShanXi, China |
| V271 | 112897 | QinLi514 | ShanXi, China |
| V141 | 112924 | YongJi1Hao | ShanXi, China |
| V142 | 112925 | YongJi2Hao | ShanXi, China |
| V60 | New lines | NangFenDaTao | ShanXi, China |
| V29 | 110014 | MianYang73-39 | Sichuan, China |
| V30 | 110821 | QingKang1Hao | Sichuan, China |
| V39 | 111593 | ChuanGaoYiFen58 | Sichuan, China |
| V57 | 113374 | KangSanXingDaTao | Sichuan, China |
| V27 | 111015 | KuiTun80-2056W(XinLuZao-3Hao) | Xijiang, China |
| V328 | 111517 | BaZhou7416 | Xijiang, China |
| V33 | 111525 | Tu188 | Xijiang, China |
| V34 | 112227 | Tu83-161 | Xijiang, China |
| V249 | 112387 | KuChe96518 | Xijiang, China |
| V11 | 112432 | KuCheT94-1 | Xijiang, China |
| V239 | 112435 | KuCheT94-4 | Xijiang, China |
| V12 | 112437 | KuCheT94-6 | Xijiang, China |
| V233 | 112439 | KuChe93551 | Xijiang, China |
| V87 | 112596 | KuChe96486 | Xijiang, China |
| V43 | 112647 | KuiTunXi96-167 | Xijiang, China |
| V2 | 112889 | BaZhou5628 | Xijiang, China |
| V31 | 140182 | Sha24-3 | Xijiang, China |
| V53 | New lines | S-050019 | Xijiang, China |
| V54 | New lines | S-050031 | Xijiang, China |
| V283 | New lines | 0102X-10-1 | Xijiang, China |
| V1 | 110452 | 70-29-5 | Yunnan, China |
| V66 | 110723 | DaLingMian | Zhaoyang, China |
| V256 | 131391 | Cook 310-5110 | Australia |
| V191 | 131628 | Ao152 | Australia |
| V192 | 131632 | AoC | Australia |
| V193 | 131643 | AoL23/757 | Australia |
| V179 | 131063 | LAPAR45 | Brazil |
| V183 | 130423 | ZhaDeMian | Chad |
| V225 | 130440 | ZhaDe3Hao | Chad |
| V186 | 130267 | L142-9 | Cote d'ivoire |
| V182 | 130403 | Europe 40 | Europe |
| V178 | 140246 | HG-BR-8 | Europe |
| V180 | 130164 | PAR-51 | Pakistan |
| V294 | 131368 | M11 | Pakistan |
| V32 | 130541 | USSR8908 | Russia |
| V46 | 131251 | USSR21Xi (91-133) | Russia |
| V219 | 131292 | USSR91Xi | Russia |
| V230 | 131309 | USSR116Xi | Russia |
| V25 | 131509 | AnJiYan6Hao | Russia |
| V181 | 130233 | Sudan2 | Sudan |
| V220 | 130391 | BPA68 | Uganda |
| V250 | 111530 | Bu3363 | US |
| V209 | 112566 | DP33B | US |
| V184 | 130237 | Acala SJ-1 | US |
| V297 | 130238 | AcalaSJ-1-9 | US |
| V173 | 130241 | Acala SJ-4 | US |
| V300 | 130242 | Acala SJ-5 | US |
| V216 | 130261 | BELLSIRO | US |
| V291 | 130294 | LineF | US |
| V168 | 130300 | AC239 | US |
| V167 | 130543 | USSR8911 | US |
| V169 | 130581 | M-8124-1159 | US |
| V280 | 130721 | PD6186 | US |
| V240 | 130797 | FB20 | US |
| V171 | 130831 | Mei8123 | US |
| V263 | 130836 | Upland | US |
| V112 | 130838 | Mei28114-313 | US |
| V166 | 130841 | MeiD#1 | US |
| V164 | 130866 | Miscot78-27 | US |
| V109 | 130867 | Miscot7803-52 | US |
| V174 | 130939 | Arcot-1 | US |
| V157 | 130943 | Arcot438 | US |
| V156 | 130944 | Arcot436 | US |
| V155 | 130947 | Arcot402bne | US |
| V158 | 130951 | Coker139 | US |
| V107 | 130961 | DES926 | US |
| V224 | 130974 | GP67 | US |
| V84 | 130977 | GP70 | US |
| V161 | 130989 | GP83 | US |
| V108 | 130998 | GP93 | US |
| V162 | 131000 | GP95 | US |
| V159 | 131039 | GP137 | US |
| V160 | 131040 | GP138 | US |
| V110 | 131076 | N73DeltapineNGF | US |
| V111 | 131081 | N74-250 | US |
| V63 | 131164 | Acala (DaLing)B | US |
| V208 | 131166 | Acala 927 | US |
| V253 | 131395 | DP2156 | US |
| V165 | 131408 | MM-2 | US |
| V207 | 131439 | UA887(You) | US |
| V213 | 131441 | ST474 | US |
| V245 | 131613 | DP410B | US |
| V170 | 131662 | AoSiv2 | US |
| V113 | 131664 | Qik | US |
| V196 | 131739 | MeiF-18 | US |
| V115 | 131747 | MeiG-84 | US |
| V197 | 131847 | MeiF-19 | US |
| V114 | 131848 | MeiG-82 | US |
| V330 | New lines | Acala1517-2 | US |
| V254 | 131468 | r-3149 | Vietnam |
| V255 | 131470 | r4136 | Vietnam |

**Note**: New lines were new breeding lines and genetic stocks bred by Institute of Cotton Research of Chinese Academy of Agricultural Sciences (ICR, CAAS) in recent years.
